# Orthodontic treatment in periodontally compromised patients: a systematic review

**DOI:** 10.1007/s00784-022-04822-1

**Published:** 2022-12-11

**Authors:** Christina Erbe, Sarah Heger, Adrian Kasaj, Manfred Berres, Heinrich Wehrbein

**Affiliations:** 1 Department of Orthodontics, University Medical Centre of the Johannes Gutenberg University, Augustusplatz 2, 55131 Mainz, Germany; 2 Department of Periodontology and Operative Dentistry, University Medical Centre of the Johannes Gutenberg University, Mainz, Germany; 3 Institute for Medical Biostatistics, Epidemiology and Informatics (IMBEI), University Medical Centre of the Johannes Gutenberg-University, Mainz, Germany

**Keywords:** Periodontitis, Aggressive periodontitis, Chronic periodontitis, Orthodontic treatment, Infrabony defects

## Abstract

**Objectives:**

The aim of this systematic review was to examine the literature on aggressive and chronic periodontitis and orthodontics to clarify the therapy-relevant aspects of orthodontic treatment with altered biomechanics in periodontally compromised dentition.

**Materials and methods:**

Literature searches were conducted in the electronic databases “PubMed” and “DIMDI” using the keywords “aggressive periodontitis AND ortho*,” “aggressive periodontitis AND orthodontics,” “chronic periodontitis AND ortho*,” and “chronic periodontitis AND orthodontics” for the publication period from January 1990 to July 2022. In addition, a manual search was carried out in the selected trade journals “Community Dental Health,” “European Journal of Oral Sciences,” and “Parodontologie.” Human clinical trials were included, whereas animal experimental studies, case reports, and reviews were generally excluded. The appropriate studies were selected, and the relevant data was tabulated according to different parameters, regarding the study design, the study structure, and the conduct of the study.

**Results:**

A total of 1067 articles were found in the preliminary electronic search. The manual search and review of all related bibliographies resulted in an additional 1591 hits. After the first screening, 43 articles were classified as potentially relevant and reviewed in their original form. After the suitability test, 5 studies with a total of 366 participants were included in the final evaluation. These included one randomized controlled trial and four low-evidence intervention studies. The studies were conducted in two university hospitals and three private practices. All participants underwent scaling and root plaining and periodontal surgery before the orthodontic treatment started. Mean probing pocket depth reduction before and after the interdisciplinary treatment was analyzed in all the included studies; mean difference in clinical attachment level in four of the studies was also included. All participants were enrolled in a continuous recall system. In all studies, orthodontic therapy in periodontally compromised patients improved function and esthetics, resulting in lower probing depths and clinical attachment gains.

**Conclusions:**

Orthodontic treatment can be used for patients with reduced periodontal support to stabilize clinical findings and improve function and esthetics. The prerequisite for this is a profound knowledge of altered biomechanics and an adapted interdisciplinary treatment approach. Due to the large heterogeneity of the included studies and their limited methodological quality, the results obtained in this review must be considered critically. Further randomized controlled long-term studies with comparable study designs are necessary to obtain reliable and reproducible treatment results.

**Clinical relevance:**

Patients with periodontal impairment can be successfully treated with orthodontics as part of interdisciplinary therapy. Orthodontic treatment has no negative impact on the periodontium; if minimal, controlled forces are used under non-inflammatory conditions.

## Introduction

The need of appropriate orthodontic appliances for the challenging treatment of adult patients has increased considerably, especially within the last six decades [1, 2]. The main reasons for the increasing demand for orthodontic treatment among adults are beauty ideals on the one hand, and the development of almost invisible appliances on the other. According to a survey by the American Association of Orthodontists® (AAO), the number of adult patients in the USA and Canada increased by 16% between 2012 and 2014. Accordingly, 27% of all orthodontically treated patients in these two nations were at least 18 years old [3].

Since a flawless appearance is today associated with better social and professional opportunities and increased self-confidence, more and more adults place high expectations on the abilities of orthodontists [4–6]. The treatment of this patient group in particular is proving to be highly demanding, often requiring interdisciplinary cooperation with periodontists, restorative dentists, implantologists, and maxillofacial surgeons.

Every orthodontic treatment is based on the interaction of teeth with their respective periodontium. In adults, however, the conditions have changed. Periodontopathies, which increase with age, lead to a destruction of the periodontal supporting tissue. This results in a smaller reaction zone between the root surface and the convertible alveolar bone. The resistance center of the affected teeth also shifts further towards apical due to the resorption of the alveolar ridge. These changes result in a greater deflection of the teeth [7] when force is applied and the risk of unwanted tilting and root resorption increases. Careful treatment planning must ensure inflammation-free periodontal conditions and take into account changes in biomechanics. A structured interdisciplinary treatment approach that considers the individual therapeutic needs and possibilities is decisive for the long-term therapeutic success.

## Materials and methods

### Search strategy

The literature search was carried out within the electronic databases “Pubmed” and “DIMDI.” The search was carried out with previously defined search keys for the publication period from January 1990 to July 2022:

1. aggressive periodontitis AND ortho*
2. aggressive periodontitis AND orthodontics
3. chronic periodontitis AND ortho*
4. chronic periodontitis AND orthodontics

In addition, the journals “Community Dental Health,” “European Journal of Oral Sciences,” and “Parodontologie” were searched by hand.

### Study design

The literature search comprised randomized controlled trials (RCT), cohort studies, case–control studies, and cross-sectional studies. Animal experimental studies, case reports, and reviews were generally excluded.

### Population

Only studies with systemically healthy patients were included.

### Language

The searches were confined to publications in German or English.

The titles and short descriptions of the publications identified by the database and hand search were analyzed, and the full text examined to determine whether it matched the search criteria. If a match with the keywords could not be clearly identified from the abstract, the corresponding original work was requested and checked. Finally, the bibliographies of the present full texts were reviewed in the same way.

The analysis of all publications was subject to a complex staggered search scheme.

## Results

### Literature search

**Fig. 1:**
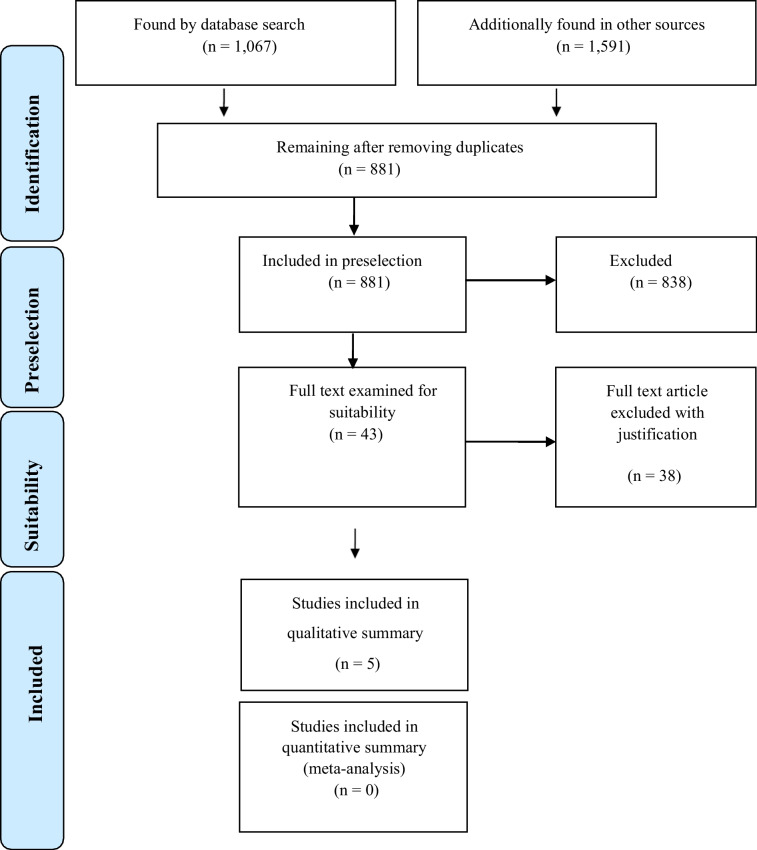
PRISMA flow chart of literature search [8]

The electronic literature search yielded 1067 publications (Fig. 1). The manual search and review of all related bibliographies resulted in an additional 1591 hits. Of these, 881 publications remained after the exclusion of duplicates. Following screening, 43 articles were classified as potentially relevant and the originals were reviewed. As a result, 6 studies were excluded whose thematic focus differed from the research question. 2 studies were excluded (Cardaropoli D et al. 2001, Re S et al. 2004) as they were identical to two studies, which were already included in this review (Corrente G et al. 2003, Cardaropoli D et al. 2004). 28 publications were not taken into account due to deficiencies in content. Thus, some of these articles did not provide any evaluable information about material and methods or results of the investigation, especially the periodontal parameters are missing. In two publications, periodontal pre-treatment and orthodontic extrusion served the sole purpose of implant site preparation; i.e., periodontally compromised teeth were extracted prior to implant placement. This treatment concept contradicts the question posed in this paper.

Finally, five studies were analyzed for this systematic review. Table 1 lists these studies with their characteristic features regarding the study design, the study structure, and the conduct of the study. The studies differ a lot in the statistical heterogeneity:

**Table 1:** Overview of the included studies

Study characteristics	Ogihara S, Wang HL (2010)	Re S et al. (2000)	Ghezzi C et al. (2008)	Cardaropoli D et al. (2004)	Corrente G et al. (2003)
Study design	Randomized controlled trial	Intervention study	Intervention study	Intervention study	Intervention study
Follow-up	1 year	Division of patients into 5 groups with different follow-up intervals: 1) 64 Pat → 2a 1) 62 Pat → 4a 3) 59 Pat → 6a 4) 66 Pat → 10a 5) 16 Pat → 12a	n.a	1 year	-
Country	Japan	Italy	Italy	Italy	Italy
Institution	Private practice for periodontology, Tokyo	University dental clinic and private practice, Turin	Dental clinics of the Universities of Milan and Pavia, private practices in Saronno and Pessano	Private practice, Turin	Private practice, Turin
Number of cases	47	267	14	28	10
Age	Ø53 ± 10.7 years	Ø44 ± 7.9 years	Not specified	Ø45 ± 7.8 years	33–53 years
Inclusion criteria	1) Radiologically confirmed intrabony defect ≥ 6 mm 2) Completed systematic periodontitis therapy 3) Chronic periodontitis	pathological tooth migration in the front	1) Presence of an intrabony defect ≥ 6 mm 2) Completed periodontitis pre-treatment with scaling and root planning (SRP) 3) Pathological tooth migration with diastema 4) ≥ 21a	1) Radiologically confirmed intrabony defect ≥ 6 mm 2) Completed periodontitis pre-treatment with SRP 3) Pathological tooth migration with diastema 4) Chronic periodontitis	1) Radiologically confirmed intrabony defect ≥ 6 mm 2) Completed periodontitis pre-treatment with SRP 3) Pathological tooth migration 4) ≥ 1 mm extrusion on upper central incisor
Exclusion criteria	1) Smokers 2) Pregnant women and nursing mothers	-	1) Smokers 2) General diseases 3) Pregnant women and nursing mothers	1) General diseases 2) Regular medication	1) General diseases 2) Regular medication
Investigation parameters	1) Probing depth 2) Clinical attachment level 3) “Open probing attachment level” 4) Proportion of defect healing (%)	1) Probing depth 2) Bleeding on probing (% BOP)	1) Probing depth 2) Clinical attachment level 3) Recessions 4) Papilla Presence Index (PPI)	1) Probing depth 2) Clinical attachment level 3) PPI 4) Distance between contact point-limbus alveolaris) Periodontal biotype	1) Probing depth 2) Clinical attachment level 3) Vertical (TD-BC) and horizontal (BC-TD) expansion of the intrabony defect
Pretreatment	SRP Elimination of occlusal trauma	SRP	SRP	SRP	SRP
Periodontal Surgery	Regenerative periodontitis surgery: Emdogain, demineralized freeze dried bone allograft (DFDBA)	Modified Widman cloth and open SRP	1) Modified or simplified papilla preservation cloth 2) Enamel matrix proteins or bone graft + membrane	Mucoperiosteal flap, open SRP	Mucoperiosteal flap, open SRP
Post-surgical measures	Cleaning prohibition 7 d, antibiosis 4d as well as weekly control: plaque removal, chlorhexidine (CHX) rinsing, remotivation	no details	Antibiosis 6d, 0.2%-CHX rinsing first 15 d, then 0.12%-CHX- rinsing 14 d	no details	Cleaning prohibition and 0.2% CHX rinsing 7–10 d postoperative
Study characteristics	Ogihara S, Wang HL 2010	Re S et al. 2000	Ghezzi C et al. 2008	Cardaropoli D et al. 2004	Corrente G et al. 2003
Orthodontic technique	no details	Segmented arch technique	Straight-wire technique	Segmented arch technique	Segmented arch technique
Orthodontic movement	1) Extrusion 2) Retention	intrusion	Intrusion	1) Intrusion 2) Uprighting	1) Intrusion 2) Uprighting
Orthodontic appliance	extrusion mechanics	Multibracket appliance (MBA)	MBA	MBA	MBA
Orthodontic force	60–120 g	10–15 g	Not specified	10–15 g	10–15 g
Duration of orthodontic treatment	1 month	10 ± 3.0 months	Not specified	12 months	10 ± 2.6 months
Orthodontic retention	Temporary wire fixation (4 weeks)	Fixed	Fixed	Fixed	Fixed
Supportive Periodontitis Therapy (SPT)	on a weekly basis	Quarterly	Monthly	Quarterly	Quarterly
Re-evaluation	1) 6 months postoperative 2) 12 months postoperative	1) Postoperative 2) Follow-up	1) After periodontitis pretreatment 2) 1a postoperative 3) According to orthodontics	1) Before periodontal surgery 2) According to orthodontics 3) 1a Posttherapeutic	After orthodontic treatment
Level of Evidence (based on the Oxford Centre of Evidence Based Medicine	1b	4	4	4	4
GRADE Rating	High	Moderate	Low	Low	Low

Methodological heterogeneity:

- 1 RCT, 1 intervention study with two intervention groups, 3 intervention studies without control group.
- From *n* = 267 to *n* = 10, significantly different case numbers
- Significantly different follow-up intervals or no follow-up at all
- Timing of outcome measurement very different, partly after each intervention (PA pretreatment, PA surgery, orthodontics, follow-up), partly only before-after

Clinical heterogeneity:

- Variability of participants:
  • minimal ethnic distribution, studies only from Japan and Italy
  • predominantly middle-aged patients
  • type of periodontitis not clearly classified
  • compliance of patients partly documented via plaque score, partly no info
  • different teeth treated, partly molars, partly anterior teeth
- Type and time of outcome measurements:
  • different clinical parameters collected
  • scope and type of diagnostic measures vary greatly
  • timing of measurements varies widely, partly after each intervention, partly only before-after
  • measurement partly based on study models, radiographs, partly clinical
  • measurement partly performed by one practitioner, partly by two, partly no info at all
  • length of follow-up varies
- Intervention characteristics:
  • type of PA pretreatment, surgical (with or without bone substitutes, with or without antibiosis) vs.non-surgical
  • type of orthodontic movement: Intrusion, extrusion
  • type of orthodontic appliance
  • applied orthodontic forces vary
  • duration of orthodontic treatment
  • recall interval varies

### Descriptive presentation of the included studies

The five studies under discussion included one randomized controlled trial [9] and four intervention studies [10–13] with a total of 366 adult patients (Table 1). Among them 75 patients suffered from chronic periodontitis [9, 13]. The authors of the remaining studies did not define the type of periodontitis, only describing it as severe or advanced periodontitis. All patients were treated using an interdisciplinary approach. Periodontal therapy was performed in all cases by means of supragingival and subgingival scaling and root plaining (SRP) prior to orthodontic treatment. Four studies only included patients with a vertical bony defect of ≥ 6 mm on one tooth [9, 11–13]. All studies included do not consider the type of malocclusion as well as crowding, yet several studies describe only or use as further inclusion criterion the pathological migration of teeth and the associated development of diastema due to periodontal disease [9–14]. Smokers were excluded in four studies [9, 11–13]. Re et al. did not provide any information in this respect [10]. Except for 128 patients [10], flap surgery was performed in all patients to gain visual access to the root surfaces and alveolar bone. In 61 patients [9, 11], enamel matrix proteins or bone substitute materials were used. The teeth of 47 patients were treated with an extrusion mechanism [9]; all others were treated with a multibracket appliance (MBA) for the intrusion of the teeth. All patients regularly received professional tooth cleaning and oral hygiene instructions as part of periodontal therapy. The selected studies differed considerably regarding the orthodontic technique, the force used, and the duration of treatment. After completion of the orthodontic therapy, the patients — except for the patients participating in the study by Ogihara and Wang [9] — were treated with fixed retainers for long-term stabilization. In two studies, no information was provided regarding the follow-up period. In two other studies, follow-up took place after 12 months [9, 12]. Re et al. divided their patients into five different follow-up groups (2a, 4a, 6a, 10a, 12a) [10].

### Results of orthodontic treatment on periodontal status

The following results were obtained from a total of 366 patients included in this systematic review. Among them, 343 patients from five studies received orthodontic treatment. 23 patients in one study served as control groups and were not treated orthodontically [9].

The periodontally compromised dentition was not negatively affected by orthodontic treatment in any of the studies under discussion. On the contrary, the interdisciplinary therapy reduced the probing depths by an average of 3.31 mm with an average clinical attachment gain of 5.28 mm. These values are total mean values calculated from the mean values of the included studies by weighting the respective number of cases.

**Fig. 2:**
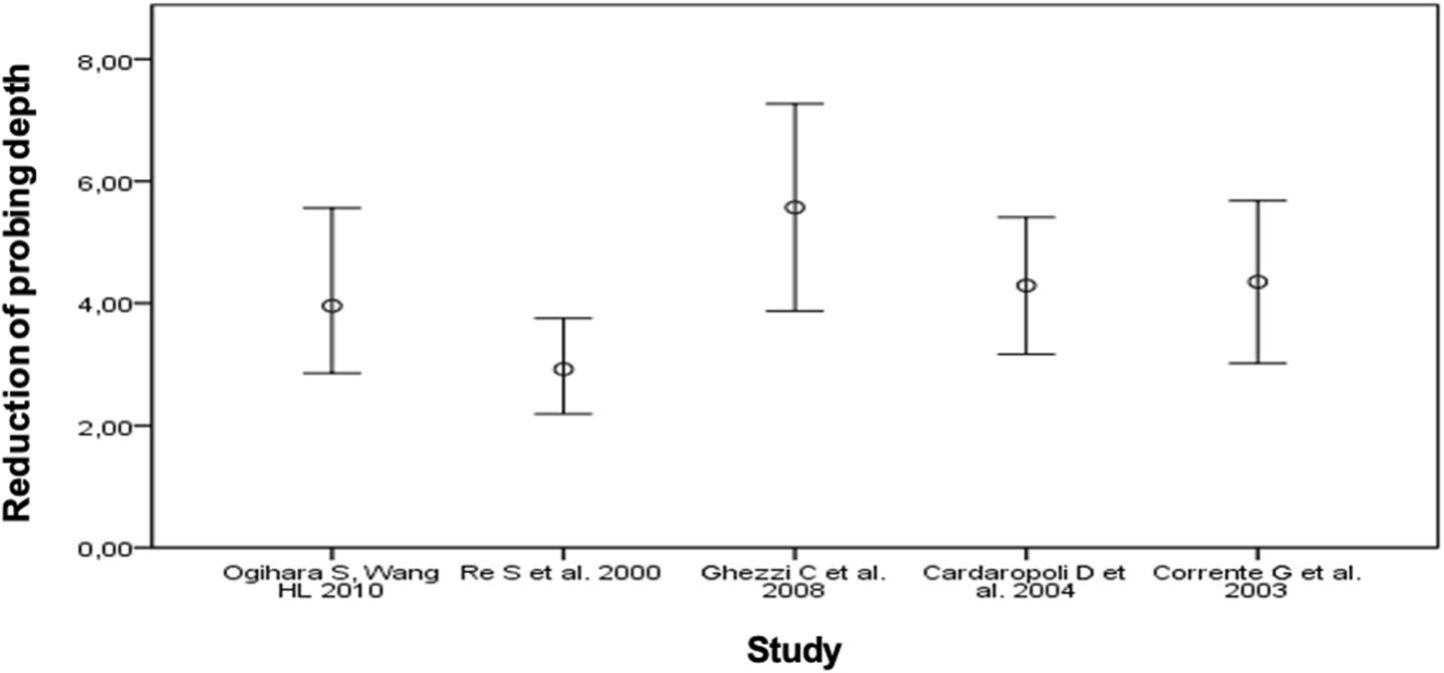
Mean with error bars depicting 1 standard deviation to describe the reduction of probing depths (mm) by periodontological orthodontic therapy

**Fig. 3:**
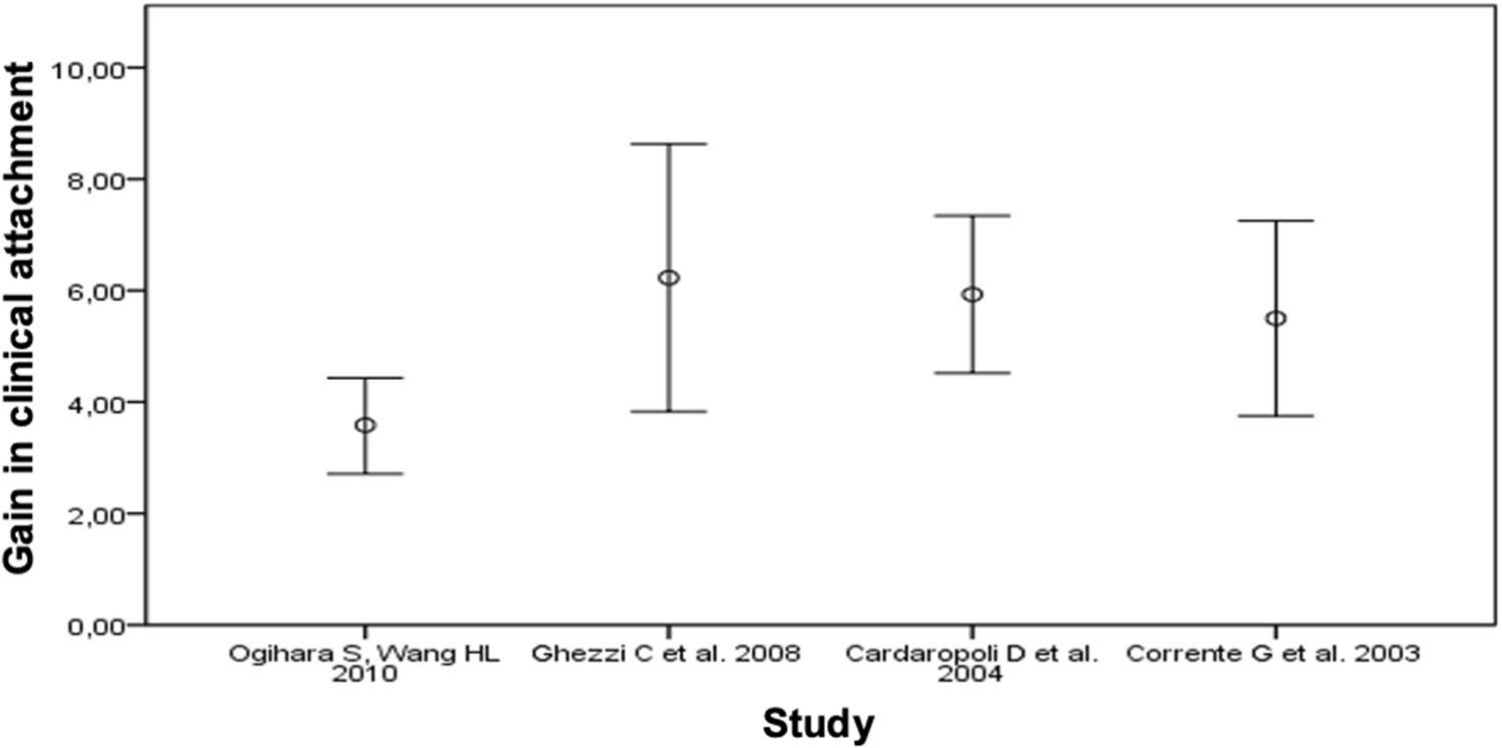
Mean with error bars depicting 1 standard deviation to describe the attachment gain (mm) through periodontological orthodontic therapy

The following figures show the decrease in probing depths (Fig. 2) and clinical attachment gains (Fig. 3) as a result of interdisciplinary therapy. The respective mean values and standard deviations are shown.

For Ogihara and Wang [9], periodontal orthodontic treatment in the intervention group resulted in ΔPPD = 4.2 ± 1.35 mm. In the study by Re et al. [10], the probing depths were reduced by an average of 3 mm. This study has by far the largest number of cases. ΔPPD is 2.9 ± 0.67 mm in the group of 128 non-surgically treated patients and 3.0 ± 0.78 mm in 129 patients who underwent open flap debridement. Ghezzi et al. [11] found a significantly larger decrease of the mean probing depth of 5.6 mm. In the studies by Cardaropoli et al. [12] and Corrente et al. [13], the mean probing depth was reduced in the course of treatment by approximately the same amount, namely, by 4.3 ± 1.12 mm and 4.4 ± 1.33 mm, respectively.

Re et al. [8] did not provide any information on clinical attachment level (CAL). While in the remaining three studies of evidence class IIc, the mean attachment gains were grouped fairly closely around a value of 6 mm, the measurement results of the patients with Ogihara and Wang [9] deviated significantly from this. In the intervention group, the CAL decreased by 3.7 ± 0.76 mm. The values of the control group are in brackets, since these patients did not receive orthodontic treatment. The diagram shows that the standard deviation is lowest in the study of Ogihara and Wang [9], so the individual values of the patients are the least widely distributed.

## Discussion

The aim of this comprehensive literature review was to clarify the therapy-relevant aspects of orthodontic treatment with altered biomechanics in the periodontally compromised dentition. Even though extensive attempts were made to collect information relating to the subject, the results of this review should be evaluated critically. The main reason for this is that both the number of relevant publications and the methodological quality of the studies are severely limited. Only one study is randomized and distributed with a control group [9]. In addition, the case numbers of the individual studies are very small and the follow-up period short, so that the long-term effect of the intervention cannot be estimated. The bias risk is correspondingly high.

This review summarizes the findings of the analyzed articles. Although a new classification of periodontal diseases has been introduced by Carton et al. [15] in 2018, this systematic review used the 1999 Classification system by Armitage et al. [16] in 1999, as most studies so far are based on this classification. The orthodontic treatment of periodontitis patients seems to have a positive effect on the affected periodontium both functionally and aesthetically. There is consensus in the literature that the establishment and maintenance of an inflammation-free periodontal condition must be ensured during orthodontic therapy and beyond. Only a regular plaque check with oral hygiene instructions and professional tooth cleaning prevents the orthodontic forces from having a negative effect on the already impaired periodontium. As early as 1977, Ericsson et al. [17] were able to show in an animal study in five Beagle dogs that tilting and intruding forces in the periodontally impaired but plaque-free periodontium does not cause infrabony defects. On the other hand, the same movements in plaque accumulated teeth caused a displacement of the supragingival plaque to subgingival, which favors a subsequent attachment loss [17]. Irregular recall during orthodontic therapy is associated with a significantly higher rate of tooth loss [18, 19]. Accordingly, all supportive periodontal therapy (SPT) patients were involved in regular follow-ups.

In addition to retraction and up righting, intrusion and extrusion play a central role in periodontitis patients and have been examined scientifically in detail. In various animal studies, the intrusion of teeth with iatrogenically induced attachment loss led to bone and cement neoplasms in the absence of inflammation [20–22]. The findings from these publications formed the basis for the later orthodontic treatment of periodontitis patients. Intrusion reduced the probing depths of migrated and extruded incisors and significantly improved the marginal bone level [14].

The use of minimal, continuous force is critical for the controlled movement of periodontally compromised teeth. As early as 1989, Melsen et al. achieved the most favorable results with orthodontic forces of 5–15 g/tooth in a comparative study using different methods of anterior tooth intrusion [23]. In patients with advanced chronic periodontitis, subcrestal defects were almost completely eliminated, interdental papillae were restored, and probing depths were reduced [14, 24, 25]. This is in line with more recent systematic reviews on this topic [26, 27].

An “optimal force” ensures fast tooth movement combined with the lowest possible discomfort for the patient and minimal damage to the surrounding tissue [28]. The position of the resistance center is decisive for this. If the attachment is lost, the resistance center of the tooth shifts to apical. As a result, orthodontic forces must be reduced in order to avoid uncontrolled tooth tilting. Bone resorption and slower bone remodeling also reduce the anchoring quality of natural teeth. In this case, the reciprocal forces generated by each tooth movement can be less easily absorbed by adjacent teeth, which results in undesirable side effects [29]. The use of skeletal anchoring elements makes orthodontic treatment possible even in these complex cases. The method is also barely visible and meets the high aesthetic demands of adult patients. Wehrbein et al. were among the first to investigate the resilience and stability of palatal implants (Orthosystem Straumann®, Straumann® AG, Basel, Switzerland). This sagittally centered titanium implant osseointegrated in the anterior palate region has proven its worth in the treatment of patients with various malocclusions [30–32]. Histologically, palatal implants show good osseointegration that ensures positionally stable anchorage throughout the period of orthodontic treatment [33, 34]. According to a scientific statement by the German Society for Orthodontics (Deutsche Gesellschaft für Kieferorthopädie e.V., DGKFO), palatal implants and cortex screws can be successfully used in a variety of orthodontic treatment tasks and provide reliable results. These anchoring elements are especially indicated in situations with reduced anchoring quality of the natural dentition, as it is common in periodontally impaired patients with extruded teeth, advanced bone resorption and tooth loss [35]. To date, however, no studies have investigated the skeletal anchoring of MBAs in periodontitis patients. Only isolated case reports document the clinical benefit [36, 37].

In addition to tooth intrusion, forced extrusion has a firm place in the orthodontic treatment of periodontitis patients. They are used for isolated intraosseous defects. These can have several causes: uncontrolled tooth tilting and occlusal trauma, but also local irritation factors such as insufficient restorations, impacted food remains or plaque. Since the neighboring teeth are usually periodontally intact, resective measures are prohibited in these cases. Depending on the morphology of the defect, regenerative methods also have only a limited effect here. Orthodontic extrusion is indicated especially for single or double-walled subcrestal defects. In this way, infrabony defects can be corrected, periodontal pockets reduced and associated gingivitis eliminated [38].

The choice of orthodontic appliance is based on the biomechanics of the findings. In most cases, fixed appliances are indicated to correct tooth misalignments in all three spatial planes. This is reflected in the publications included — MBAs were used in all patients. However, adults with a high aesthetic awareness often reject conventional MBAs. Aligners are a new generation of removable orthodontic devices that are increasingly used in adult treatment. The Invisalign® technique (Align Technology, Santa Clara, USA), which has been established for several years, uses a series of these transparent plastic splints to achieve an end position previously planned on a three-dimensional model [39]. Bite position corrections and extensive three-dimensional movements, however, cannot be implemented with aligner technique alone and still require the use of fixed appliances [40, 41]. As the splints can be removed and thus cleaned easily, they are periodontally hygienically favorable. Clinically, this property is reflected in a significantly better plaque index [42] and lower inflammation values compared to fixed appliances [43–45]. Orthodontic treatment with MBAs usually only leads to temporary impairment of periodontal structures in periodontally healthy patients [46]. However, these results must be critically questioned with regard to the reactions of the periodontally impaired patient. A shift of subgingival microbiota towards periodontal pathogenic anaerobic bacterial species during MBA treatment has been observed in numerous studies [47]. A clear objective in the use of fixed appliances must therefore be to prevent plaque accumulation as far as possible.

After augmentation, the early use of orthodontic forces seems to favor the reconstruction of the bone substitute material and the consolidation of the bony defect as soon as 5–10 days postoperatively [24]. Especially in this early phase, intrusive moments seem to have an advantageous effect on tissue remodeling and lead to aesthetically excellent results [14, 24, 25]. Cardaropoli et al. [25] see early intrusion of teeth as the prerequisite for natural periodontal regeneration. The elongation of the collagen fiber apparatus acts as a natural barrier that prevents apical epithelial proliferation and allows attachment gains. Convertible periodontal tissue is the prerequisite for functional and structural restoration of the periodontium. Diedrich et al. [29] as well as Juzanx and Giovannoli [48] therefore call for regenerative treatment of teeth with advanced attachment loss before they are moved orthodontically towards an infrabony defect. This therapy concept has been successfully implemented in case reports [49–53]. Evidence-based research proves that guided tissue regeneration and the use of enamel matrix derivatives (Emdogain®) are safe and effective regenerative methods. Their use in the treatment of infrabony defects has been proven over decades and provides predictable treatment results [54–61].

In periodontitis-damaged teeth, delayed cell remodeling often necessitates permanent retention, which can only be achieved with fixed retainers [62–64]. Flexible lingual retainers have proven their worth in these situations and are currently regarded as the gold standard. In contrast to other permanent retention measures, they allow the physiological mobility of the teeth. Unlike removable appliances, they are largely independent of the patient’s compliance. When inserting the wires, exact positioning is crucial in order to protect the surrounding soft tissue as much as possible and to enable undisturbed dynamic occlusion. Since bonded retainers promote plaque accumulation, it is essential that they show a good hygienic capability.

To date, there are no long-term studies documenting the stability of the results of periodontal orthodontic therapy [65]. However, individual case reports with long-term follow-up demonstrate that a healthy periodontal condition and stable occlusion can be permanently established. A well-thought-out interdisciplinary therapy concept with strict periodontal follow-up and excellent patient compliance are necessary prerequisites for long-term treatment success [66–70].

The most recent systematic review and meta-analysis by Martin et al. [27] examined the effects of orthodontic tooth movement on clinical attachment level (CAL) changes in stable treated adult periodontitis patients compared to non-periodontitis patients. The authors concluded that in non-periodontitis and stable treated periodontitis patients, orthodontic tooth movement had no significant impact on periodontal outcomes. This systematic review serves also as base for the clinical recommendations in the EFP S3 level guidelines for treatment of stage IV periodontitis [71]. Thus, the clinical recommendation says that in successfully treated stage IV periodontitis patients in need for orthodontic therapy, it is suggested to undertake orthodontic therapy based on evidence that orthodontic therapy has no detrimental effects on periodontal conditions in periodontitis patients with a healthy but reduced periodontium. This is in line with our findings. However, it has to be noted that in our systematic review, periodontitis patients with different levels of severity were included, evaluating orthodontic treatment in the periodontally compromised dentition more broadly.

## Conclusion

Patients with periodontal impairment can be successfully treated with orthodontics as part of interdisciplinary therapy. Provided that low controlled forces are used in non-inflammatory conditions, orthodontics will not have any negative effects on the periodontium. To obtain reliable and predictable therapy results and to formulate guidelines, further randomized controlled trials with uniform study design and regular follow-ups are necessary.

## Data Availability

The data used for analysis has been referenced in the text or tables of the paper.
